# New Insights into the Evolutionary and Genomic Landscape of Molluscum Contagiosum Virus (MCV) based on Nine MCV1 and Six MCV2 Complete Genome Sequences

**DOI:** 10.3390/v10110586

**Published:** 2018-10-26

**Authors:** Tomaž M. Zorec, Denis Kutnjak, Lea Hošnjak, Blanka Kušar, Katarina Trčko, Boštjan J. Kocjan, Yu Li, Miljenko Križmarić, Jovan Miljković, Maja Ravnikar, Mario Poljak

**Affiliations:** 1 Institute of Microbiology and Immunology, Faculty of Medicine, University of Ljubljana, Zaloška 4, SI-1000 Ljubljana, Slovenia; tomaz-mark.zorec@mf.uni-lj.si (T.M.Z.); lea.hosnjak@mf.uni-lj.si (L.H.); blanka.kusar@mf.uni-lj.si (B.K.); bostjan.kocjan@gmail.com (B.J.K.); 2 Department of Biotechnology and Systems Biology, National Institute of Biology, Večna pot 111, SI-1000 Ljubljana, Slovenia; denis.kutnjak@nib.si (D.K.); maja.ravnikar@nib.si (M.R.); 3 Department of Dermatovenereology, University Medical Centre Maribor, Ljubljanska ulica 5, SI-2000 Maribor, Slovenia; katarina.trcko@gmail.com; 4 Poxvirus and Rabies Branch, Division of High-Consequence Pathogens and Pathology, National Center for Emerging and Zoonotic Infectious Diseases, Centers for Disease Control and Prevention, 1600 Clifton Road NE, Atlanta, GA 30333, USA; lay4@cdc.gov; 5 Faculty of Medicine, University of Maribor, Taborska Ulica 6b, SI-2000 Maribor, Slovenia; miljenko.krizmaric@um.si (M.K.); miljkovicj@icloud.com (J.M.)

**Keywords:** molluscum contagiosum virus, complete genome, recombination, immune evasion, evolution, genetic landscape

## Abstract

Molluscum contagiosum virus (MCV) is the sole member of the *Molluscipoxvirus* genus and the causative agent of molluscum contagiosum (MC), a common skin disease. Although it is an important and frequent human pathogen, its genetic landscape and evolutionary history remain largely unknown. In this study, ten novel complete MCV genome sequences of the two most common MCV genotypes were determined (five MCV1 and five MCV2 sequences) and analyzed together with all MCV complete genomes previously deposited in freely accessible sequence repositories (four MCV1 and a single MCV2). In comparison to MCV1, a higher degree of nucleotide sequence conservation was observed among MCV2 genomes. Large-scale recombination events were identified in two newly assembled MCV1 genomes and one MCV2 genome. One recombination event was located in a newly identified recombinant region of the viral genome, and all previously described recombinant regions were re-identified in at least one novel MCV genome. MCV genes comprising the identified recombinant segments have been previously associated with viral interference with host T-cell and NK-cell immune responses. In conclusion, the two most common MCV genotypes emerged along divergent evolutionary pathways from a common ancestor, and the differences in the heterogeneity of MCV1 and MCV2 populations may be attributed to the strictness of the constraints imposed by the host immune response.

## 1. Introduction

Molluscum contagiosum virus (MCV) is the causative agent of molluscum contagiosum (MC) and the last known naturally circulating virus of the *Poxviridae* family with a unique tissue tropism for the human epidermis [1,2,3,4]. MC manifests in the form of small umbilicated papules, usually limited in size and number, with a typical benign clinical outcome in immunocompetent adult patients because the lesions often regress spontaneously over time [1,5]. Spontaneous regression of MC lesions is generally accompanied by signs of inflammation [5]. Less favorable clinical outcomes have been observed in children and immunocompromised patients, such as those with human immunodeficiency virus (HIV) infection or those receiving immunosuppressive therapy, in whom several larger MC lesions, which require treatment more frequently, can occur [1,4,6,7,8]. Although MC is mainly associated with cosmetic affliction, it can also lead to decreased quality of life due to severe disfiguration [1,8,9,10]. Epidemiological studies have indicated high prevalence of the disease, with a seropositivity of 23 to 30% among healthy (adult) populations in Australia and the United Kingdom [11,12], respectively, and up to 77% among HIV-positive patients in Australia [11]. Moreover, MC has been listed among the top 50 most prevalent diseases worldwide [13]. Even though MCV is an important and frequent human pathogen, data regarding its evolutionary history and molecular epidemiology are limited to profiling using restriction-fragment length polymorphisms (RFLP) and to a scarce collection of only five complete genome sequences in known sequence repositories (NCBI GenBank).

Early genomic RFLP studies suggested the existence of four major MCV genotypes enumerated MCV1–4 [14,15,16,17,18,19], with the possibility of several genotype variants [18,19]. MCV1 is the most prevalent genotype worldwide, followed by MCV2. MCV3 is universally rare, and MCV4 has so far only been found in Japan and Australia [1,16,17,18,19,20,21]. The first complete MCV genome sequence (MCV1) was assembled and annotated by Senkevich et al. in 1997 [20]. Until 2017, when three additional MCV1 isolates and the first MCV2 isolate were fully sequenced, nucleotide sequence data were only available for a limited number of MCV genes likely due to the length of MCV genomes (approximately 190,000 nucleotides (nt)). Therefore, MCV molecular assays were mostly based on short sequence fragments of the MC021L gene and allowed differentiation only between MCV1 and MCV2 through sequencing or quantitative PCR (qPCR) [21,22,23,24]. Due to the lack of nucleotide sequence data of genotypes other than MCV1 and MCV2, genomic RFLP analyses [16,18,19] remain the only method for identification of genotypes MCV3 and MCV4.

MCV immune evasion strategies and the involved viral genes have been comprehensively reviewed by Shisler [4]. A recent study of the MCV1 transcriptome [25] has consolidated most gene predictions provided by Senkevich et al. [20], and López-Bueno et al. [26] generated the first complete genome sequence of MCV2, suggesting divergent evolutionary pathways of the two main MCV genotypes and indicating the possibility of recombination events.

In this study, 10 novel complete MCV genomes (five MCV1 and five MCV2) were sequenced, assembled, and annotated. With newly generated data and complete genomes of five MCV isolates sequenced previously (four MCV1 and a single MCV2), we established the most robust database to date for studying the evolutionary and genetic landscapes of MCV, specifically the two most common genotypes: MCV1 and MCV2. In addition, our database has made possible the first investigation of the genomic diversity of MCV2 as well as the most comprehensive study of MCV recombination events.

## 2. Materials and Methods

A total of 15 complete MCV genome sequences were interrogated in this study (Table 1). To the best of our knowledge, 14 out of 15 MCV sequences were obtained from single MC lesions of individual patients. For the sequence with GenBank accession number (acc. no.) U60315 [2], it is unclear whether it was obtained from a single or several MC lesions of individual or several patients (Table 1). Out of 15 MCV complete genomes studied, five were readily available in GenBank (Table 1, Nos. 1–4 and 10), and the remaining 10 MCV sequences (Table 1, Nos. 5–9 and 11–15) were generated for the purpose of this study by next-generation sequencing (NGS), followed by de novo assemblies. To generate complete MCV genome sequences, ten DNA isolates were selected from the collection of 188 isolates obtained from the same number of Slovenian patients with histologically and virologically confirmed MC [27]. Original DNA extraction was performed from MC tissues using the QIAmp DNA Mini Kit (Qiagen, Hilden, Germany). Ten complete newly assembled annotated MCV genome sequences were submitted to the GenBank database under acc. Nos. MH320547–MH320556 (Table 1). 

### 2.1. Ethical Approval

This study was approved by the Slovenian National Medical Ethics Committee (approval No. 0120-168/2017-3 KME 47/04/17).

### 2.2. Selection of Clinical DNA Isolates for NGS

Ten MCV isolates to be sequenced were chosen based on phylogenetic clustering of *MC079R* and *MC148R* gene fragments obtained from 85 and 57 samples (fragment lengths: 487 and 301 nt), respectively, in a pilot experiment. MCV genotypes were initially identified using qPCR based on amplification of the *MC021L* region, as described previously [24]. To include samples that could exhibit recombination events and capture the highest possible degree of diversity, the remaining two preliminary phylogenetic trees were examined for sequences that exhibited higher degrees of divergence and/or clustered with different MCV genotypes than in the qPCR-based MCV genotype classification of the sample [24]. Because NGS was performed as whole-genome shotgun (WGS) sequencing of clinical DNA isolates, without any enrichment of the viral fraction, only samples with viral loads of at least 1000 viral copies per single human cell, estimated by qPCR [24], were considered eligible for NGS. Finally, five MCV1 and five MCV2 samples were selected and fully sequenced; of these, three samples were sequenced only using Illumina (San Diego, CA, USA), whereas the remaining seven samples were sequenced using both Illumina and Oxford Nanopore approaches (for details, see Table 1).

### 2.3. Sequencing

#### 2.3.1. Illumina Short-Read Sequencing

Sequencing libraries for samples Nos. 8 and 9 were prepared at Otogenetics (Otogenetics Corporation Inc., Norcross, GA, USA) directly from DNA isolates, using the Nextera DNA Library Prep Kit (Illumina), and sequenced in paired-end mode (2 × 150 nt and 2 × 250 nt) on the HiSeq2000 platform (Illumina).

The remaining eight samples (Nos. 5–7 and 11–15) were first processed with RNAse A (Qiagen, Hilden, Germany), according to the manufacturer’s instructions, followed by DNA concentration estimation on a Qubit 4 Fluorimeter platform (Thermo Fisher Scientific, Waltham, MA, USA), using the Qubit dsDNA HS Assay Kit (Thermo Fisher Scientific). All samples with an estimated DNA concentration below 5 ng/μL were further subjected to non-specific amplification, using the REPLI-g UltraFast Mini Kit (Qiagen), and treatment with the T7 Endonuclease I (New England BioLabs, Ipswich, MA, USA). Sequencing libraries were prepared at GATC (GATC Biotech, Konstanz, Germany), using the GATC Biotech in-house automatic library preparation method, and sequenced in paired-end mode (2 × 150 nt) on the HiSeq4000 platform (Illumina).

#### 2.3.2. Oxford Nanopore Technologies (ONT) Long-Read Sequencing

Seven DNA isolates used for long-read sequencing (Nos. 5, 6, 8, 9, 11, 14, and 15) were subjected to whole-genome amplification (WGA) and post-processing in the same manner as described in the previous section (Illumina short-read sequencing). ONT sequencing libraries were prepared following the whole-genome amplification protocol (version WAL_9030_v108_revD_26Jan2017). After WGA and endonuclease treatment, sequencing libraries were prepared without shearing and with end repair using a Ligation Sequencing Kit 1D (SQK-LSK108; ONT). The sequencing libraries of the samples were barcoded with the Native Barcoding Kit 1D (EXP-NBD103; ONT) to allow parallel sequencing of several samples at the same time. Barcoded libraries were sequenced in pools of three using FLO-MIN107 flow cells on a MinIon MK1b device (ONT), according to recommendations of the manufacturer. The amount of each individual library in the sequencing pool was adjusted to aim at an extrapolated minimum sequencing depth of 100× for each sample and, further, to minimize the sequencing time, the pooling was optimized based on the prior qPCR-based viral load estimates for each sample. Platform quality control experiments were performed for each new ONT flow cell and after every wash run, performed in between batch runs, with the ONT Flow Cell Wash Kit (EXP-WSH002), according to the manufacturer’s recommendations.

### 2.4. Sequence-Data Processing

#### 2.4.1. Short-Read Data Pre-Processing

Initial sequence read quality control was conducted using FastQC v0.10.1 [28], indicating anomalous base content in the first 15 nucleotides of reads in all short-read datasets, which were then trimmed using bbduk [29].

#### 2.4.2. Long-Read Data Pre-Processing

Basecalling was performed with Albacore software v2.0.2 (ONT) [30]. Sample barcode de-multiplexing and adapter trimming was carried out with Porechop [31], using a 70% barcode identity and a 5% barcode identity difference threshold.

Long-read error correction was performed with Nanocorr [32], which allows correction of long ONT reads with short Illumina reads.

#### 2.4.3. Genome Assembly

Due to the presence of host DNA and genetic material likely originating from cutaneous microflora, the sequence assemblies were performed in two steps. First a reference 21 nucleotide (nt) k-mer database, using the complete MCV genome sequences available in GenBank (samples Nos. 1, 10, and 2–4; acc. Nos. U60315, KY040274, and KY040275–KY040277, respectively), was constructed and used to fish-in reads that contained k-mers found in the reference database (positive filtering). Positively filtered data sets were then assembled, and contigs showing similarity to MCV (according to the NCBI blastn; https://blast.ncbi.nlm.nih.gov/Blast.cgi) were collected and added to the filtering reference database. Positive filtering was then repeated at a k-mer length of 27, followed by a second de novo assembly step.

Within the overall genome assembly workflow described above, various assembly strategies were tested and evaluated, including the use of various assembler software (SPAdes v3.11.1 [33], Unicycler [34], Canu [35]) with different assembler-specific parameter settings, different data subsets, and using corrected or uncorrected reads (in the case of ONT reads). In addition to de novo approaches, consensus sequences obtained through mapping NGS reads to known reference sequences were also inspected. Assembled genome models were refined based on short-read remapping using Pilon v1.22 [36]. Evaluation of assemblies was based on several metrics. First, the assembled contigs or scaffolds obtained by overlapping contigs, resembling MCV, were required to exhibit a length of approximately 180,000 to 200,000 nt and both inverted terminal repeat regions (ITR regions) were required to be nearly identical in their reverse complements. In remapping, using bwa v07.12-r1039 [37], the entire presumed MCV contig/scaffold needed to be covered with Illumina reads. Furthermore, feature response curves (FRCbam; [38]), paired-end insert-size distributions, and the numbers of gene annotations (RATT; [39]) that could be transferred from any of the available reference sequences available in GenBank (acc. Nos. KY040274–KY040277 [26] (Nos. 10, 2–4), U60315 [20] (No. 1)) were inspected.

Assemblies passing the inclusion criteria and generally receiving highest scores in different assembly evaluation tests (described above) were produced with SPAdes (with refinement; Pilon) by applying the following parameters: “-k 21,33,55,77,99,127”, “--careful”, and “--cov-cutoff auto”. In samples in which ONT reads were sequestered (Table 1), corrected ONT reads were integrated into the SPAdes assembly by setting the “--sanger” parameter. Assemblies in which both Illumina and ONT reads were used are herein termed hybrid assemblies, whereas assemblies based only on Illumina reads are termed short-read assemblies.

All hybrid assemblies produced single-contig MCV genomes, whereas in some short-read assemblies the MCV genomes were finally scaffolded according to overlap from two assembled contigs. A validity assessment of the aforementioned overlap-scaffolding procedure was carried out by comparing the hybrid and short-read assemblies in cases in which both Illumina and ONT reads were available, and the short-read assembly had to be scaffolded to obtain a complete MCV genome. Although inclusion of ONT reads did seem to affect the lengths of the ITRs, none of the remaining metrics showed deterioration of sequence assembly quality; most importantly, the number of annotations transferred did not differ between the hybrid and short-read assemblies of the same sample.

#### 2.4.4. Genome Annotation

The final annotation of complete MCV genome sequences was conducted adopting the annotation transfer methodology using GATU [40]. Annotations were transferred by merit of protein similarity of identified open reading frames (ORFs) to the genes annotated in reference sequences available in the GenBank database (No. 2 GenBank acc. No. KY040275 for MCV1 genomes, and No. 10 GenBank acc. No. KY040274 for MCV2 genomes). All unassigned ORFs were queried with the NCBI Blastp (searches were restricted to the genus *Molluscipoxvirus*). Although no new annotations could be established, in some cases an alternative ORF presented higher protein sequence similarity and alignment length than the one that had already been automatically annotated by GATU; in these cases, the more appropriate alternative was applied to the annotation. All annotated genes that indicated less than 60% protein similarity to known MCV reference genes and genes with gaps in the alignment to the highest scoring similar protein were subjected to local re-assemblies for the regions in question, with a ~200 nt overhang on each side. The final protein similarity threshold for annotating a gene was set at 40%.

### 2.5. Diversity Estimation and Phylogenetic Trees

Complete MCV genome sequences were aligned with mafft v7.271 [41]. Nucleotide/protein sequences of genes that appeared in all MCV genomes (consensus genes) were further aligned with muscle v3.8.31 [42] to produce codon alignments. Pairwise *p*-distances were calculated from multiple nucleotide sequence alignments (MSA) using Mega CC 7.9.26 [43].

Phylogenetic trees were obtained with PhyML [44] using the generalized time reversible model [45] with invariable sites and four gamma categories (GTR + I + G); proportions of invariable sites and base frequencies were estimated from each MSA. For amino acid phylogenies, the JTT model [46] was used instead of the GTR. Branch support values were calculated as approximate likelihood ratio test (aLRT) supports. All phylogenetic trees were rooted using midpoint outgroups. Automation of phylogenetic analysis was carried out using the ETE3 toolkit [47].

Overall, intra- and inter-genotype diversity estimates were calculated from *p*-distance matrices, facilitated through the use of the Numpy Python module [48]. Statistical testing was carried out through the utilities provided in the Scipy module [49].

Uneven sampling of complete MCV genomes of the two MCV genotypes (9 × MCV1, 6 × MCV2) introduced a numerical bias that affected the per-sample mean distance calculation. To correct the mean per-sample *p*-distances of every MCV genome interrogated, a bootstrap-like combinatorial sub-sampling approach was utilized, termed balancing. The mean per-sample *p*-distance was calculated for every possible subset of the distance matrix that included six genomes of MCV1 and MCV2, respectively. The obtained sets of mean per-sample *p*-distances were then arithmetically averaged, yielding a corrected mean *p*-distance, which should represent a more accurate approximation of the population per-sample mean *p*-distances. It is important to note that the values of the standard deviations (*SD*) obtained through the balancing procedure no longer described the dispersion of per-sample *p*-distances, but rather the dispersion of the mean per-sample *p*-distances of each combinatorial subset.

### 2.6. Evaluation of Genome Mosaicity and Recombination

To assess the overall mosaicity of the genetic landscape of MCV, first-order linkage maps were constructed and presented as circular diagrams, linking each sample to its nearest neighboring samples according to the peak sequence similarities in the complete genome, concatenated consensus genes, and individual gene contexts. During first-order linkage map construction, only genes that included variant columns in their MSAs were considered relevant. More specifically, if the *p*-distance matrix suggested an inter-genotype link in a given gene, the entire MSA was required to contain variant columns, whereas in the case of intra-genotype links the genotype-stratified subset of the MSA had to exhibit variability. Proportions of invariable columns in the alignments were calculated using the utilities provided by the Scikit-bio Python module [50].

Codon alignments of individual gene sequences were screened for indication of recombination events between MCV genotypes using the silhouette coefficient [51], calculated based on pairwise *p*-distance information and the *MC021L*-based genotype assignment. Calculation of silhouette coefficients was facilitated through the Scikit-learn Python module [52]. The threshold value of the minimum silhouette coefficient in a gene alignment was set at 0.75, where values below the threshold indicated the possibility of recombination. Invariable gene alignments, identified by calculating proportions of variable columns in the MSA (the proportion of variable columns had to indicate a non-negative value in the overall MSA as well as in at least one of the genotype-stratified MSAs), were filtered out of this analysis. Finally, putative recombinant genes were confirmed with close inspection of their individual maximum likelihood phylogenetic trees (GTR + I + G) and by interrogation of MSAs, including the wider nucleotide sequence context of the recombinant genes (2000–5000 nt upstream and downstream), using the Recombination Detection Program 4 (RDP4; [53]). Recombination breakpoint positions were identified using the RDP, bootscan, and MaxChi methods, at the maximum *p*-value cutoff used for null hypothesis rejection of 1.2 × 10^−14^ [54].

Identified recombinant segments were screened for possible intra-sample variants. Alignments of NGS reads to recombinant segments were generated with bwa v07.12-r1039 [37], and putative variant sites were identified using Lofreq [55] and filtered according to the empirically determined threshold in alternate allele frequency of 0.1.

## 3. Results and Discussion

### 3.1. MCV Genome Assembly and Annotation

The analysis of 15 MCV genome annotations highlighted 164 MCV species-level consensus genes (consensus genes are the intersection of genes present in all known genomes of a given taxonomic unit), and 168 MCV1 and 170 MCV2 genotype-level consensus genes (Table 1). Notably, although the same 170 genes were identified in all MCV2 genomes, the number of annotated genes varied considerably among MCV1 genomes, ranging from 175 to 181 (Table 1 and Table 2).

Senkevich et al. [20] previously reported 182 MCV1 genes, with 154 genes predicted with confidence and termed likely genes. Herein, all 154 likely genes were consensually accounted for in genomes of MCV1, whereas, in accordance with the report by López-Bueno et al. [26], only 152 likely genes were identified in genomes of MCV2. The two likely genes lacking in MCV2 genomes, MC006.1R and MC144R, exhibited similar truncation and insertion/deletion patterns in all six currently known MCV2 genomes. Moreover, both missing genes in MCV2 genomes represent predicted, hypothetical, or putative proteins without known structural homologues, and to date they have not been identified as crucial for the propagation and/or survival of the virus (Table 2).

Variation in the number of annotated genes among MCV1 genomes has already been described previously (Nos. 1, 2, 3, 4; Table 2, [20,26]). In this study, the two most frequently aberrantly annotated genes, *MC001R* and *MC164L*, lie at the inner parts of the viral ITR regions and may have been missed during the annotation procedure due to relatively lower local assembly accuracy. Even though statistical approaches have been proposed and implemented for repeat-resolution in the field of de novo sequence assembly [56], the accuracy of reconstruction of repetitive regions remains challenging to assess due to ambiguous mapping of NGS sequence reads, which, by definition, mandates inherently lower read-mapping confidence/quality scores. Although the variation in number of annotated genes among MCV1 genomes could result from locally misassembled nucleotide sequence regions, it is likely that the variability is also a result of the actual diversity of the MCV1 population because all MCV2 genes were annotated very consistently across all six MCV2 genomes.

Since all MCV genomes were effectively annotated using the complete MCV1 genome sequence U60315, by merit of protein similarity, it could be speculated that MCV2 contains additional genes that may not be present in genomes of MCV1 and have so far remained unidentified. Once additional complete MCV genome sequences become available, a thorough revision of MCV gene annotation could potentially identify the presence of novel genes.

### 3.2. MCV1 and MCV2 Evolved from a Common Ancestor Along Divergent Evolutionary Pathways

**Figure 1:**
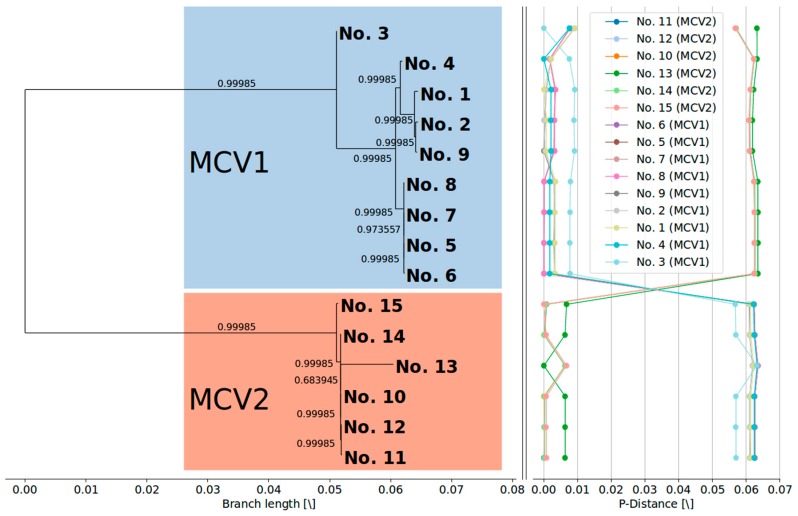
(**Left**) Maximum likelihood phylogenetic tree (GTR + I + G) with metric branch lengths and aLRT branch support values constructed based on the alignment of 15 complete MCV genome nucleotide sequences. (**Right**) Genome-to-genome *p*-distance plots, depicting a relatively large gap between the genomes of two different MCV genotypes. The phylogenetic tree was visualized using the BioPython Phylo module [57], and visualization of the pairwise *p*-distance plots was done using the Matplotlib (v2.2.2) Python module [58].

Phylogenetic clustering of complete MCV genome sequences (Figure 1) indicated a clear evolutionary divergence between the MCV1 and MCV2 genotypes: the two genotypes grouped as distinct clusters with strong aLRT branch support. Divergent evolutionary pathways of different MCV genotypes were already implied by different genomic RFLP patterns in early epidemiological studies [16,18,19]. In addition, a recent study that generated the first complete MCV2 genome [26] phylogenetically grouped the single MCV2 isolate separately from four MCV1 genomes known at that time. The results of our study are consistent with the results of previous reports and finally asserted the postulated evolutionary divergence of MCV1 and MCV2 (Figure 1). Our results indicate mean pairwise distances in the range of 1 × 10^−3^ to 1 × 10^−2^ among MCV genomes of the same genotype and 1 × 10^−2^ to 1 × 10^−1^ among MCV genomes of different genotypes. Moreover, the present data have consolidated the *MC021L*-based MCV1/MCV2 genotype differentiation [21,22,23,24].

Similarly to the findings of López-Bueno et al. [26], phylogenetic clustering of the updated complete MCV genome dataset suggests that isolate No. 3 (Table 1) forms a clearly separate lineage within the MCV1 clade (Figure 1). Moreover, the current phylogenetic tree of complete MCV genome sequences implies the existence of at least two additional lineages within the MCV1 clade beyond the divergence point of sample No. 3 from the rest of the MCV1 samples (Figure 1).

The overall mean GC content measured from the currently captured population of MCV samples is in line with the results of previous studies [2,20,26] and amounted to 0.6372 (standard deviation (*SD*) = 4.5 × 10^−3^) at the level of complete genomes and 0.6468 (*SD* = 4.4 × 10^−3^) at the level of concatenated sequences of consensus genes (Table 3). Notably, the results of our study suggest a slight, yet statistically significant, difference between MCV1 and MCV2 genomes in the underlying probability distributions of their GC content (Table 2; 2-sample Kolmogorov–Smirnov test, *p* < 0.01, group sizes: N_MCV1_ = 9, N_MCV2_ = 6), which could be a result of evolutionary divergence. According to currently available data, the GC content of complete MCV genome sequences and concatenated consensus genes, respectively, were 0.6336 (*SD* = 9.8 × 10^−4^) and 0.6421 (*SD* = 3.3 × 10^−3^) for MCV1, and 0.6425 (*SD* = 1.5 × 10^−3^) and 0.6523 (*SD* = 2.3 × 10^−4^) for MCV2, respectively.

### 3.3. Currently Available Data Suggest that MCV1 is More Diverse than MCV2

**Figure 2:**
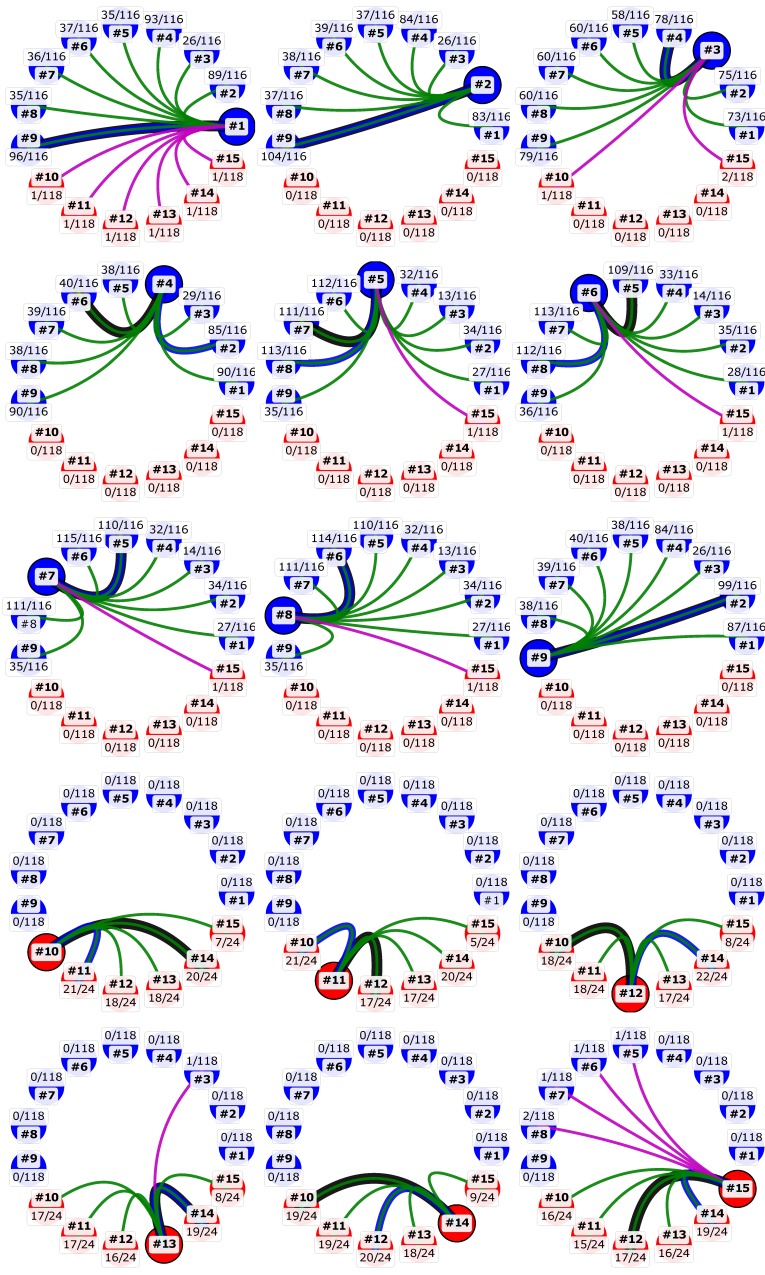
First-order linkage maps of the MCV genomes interrogated, where each genome is represented as a colored node. Nodes are colored according to the MCV genotype (blue = MCV1; red = MCV2). Edges connect MCV genomes according to their nearest neighbors based on pairwise nucleotide sequence similarities (linkage) in different contexts (colored: black, blue, red, green, and purple). Black edges connect MCV genomes according to their linkage in the complete genome alignment. Blue edges represent linkage according to concatenated alignments of consensus genes. Linkage in individual genes is represented with green (intra-genotype) and purple (inter-genotype) edges. Counts of relevant neighboring MCV genes supporting each gene edge versus MCV genes that exhibit variation in the alignment of the relevant context (intra-and inter-genotype) are shown above or below the genome identifiers. Visualization was prepared using the Matplotlib (v2.2.2) Python module [58].

Our study showed a higher degree of diversity among genomes of MCV1 in comparison to MCV2 (Figure 1, Table 3). The two intra-genotype *p*-distance samples originate from two different probability distributions (two-sample Kolmogorov–Smirnov test; *p* < 0.01; group sizes: N_MCV1_ = 72, N_MCV2_ = 30). The mean overall and mean inter-genotype *p*-distances amounted to 3.555 × 10^−2^ (*SD* = 2.957 × 10^−2^) and 6.164 × 10^−2^ (*SD* = 1.700 × 10^−3^), respectively, at the complete genome level. On the other hand, the mean intra-genotype *p*-distances among MCV1 and MCV2 were 3.740 × 10^−3^ (*SD* = 2.898 × 10^−3^) and 2.841 × 10^−3^ (*SD* = 2.738 × 10^−3^), respectively. Moreover, our results indicated that 118, 116, and (only) 24 consensus genes exhibited variation in the complete, MCV1-specific, and MCV2-specific MSAs, respectively (Figure 2), which further illustrates the relatively higher genomic diversity of the MCV1 population.

However, it is important to note that the current impression of genomic diversity of MCV and its genotypes may be biased by the somewhat limited number of MCV1 and MCV2 complete genomes available. Further studies, which would include wider samplings of complete MCV genome sequences, are needed to confirm and/or modify the current observations regarding differences in genomic diversities of individual MCV genotypes.

### 3.4. Recombination Explains Inter-Genotype Mosaicity of MCV and Anomalously High Dissimilarities Among Genes of the Same MCV Genotype

Recombination had been previously reported between different species of poxviruses [59,60,61,62], within the same poxvirus species [63,64,65], and between different genotypes of MCV [26]. Identification of inter-genotype recombination within MCV would mandate that, at some point in time, at least two different MCV genotypes existed in the same MC lesion, thereby confirming the prospect of concurrent infection with different MCV genotypes. On the other hand, the observed high viral loads (Table 1) could also facilitate the emergence of quasi-species within a single MC lesion. It is important to note that most sequencing techniques, which do not involve amplification and sequencing of viral DNA from individual viral particles, could potentially misidentify the presence of different strains (genotypes) of the virus for recombination.

Examination of the first-order linkage maps (Figure 2) indicated a high degree of genomic mosaicity among MCV genomes: although a given pair of MCV samples may exhibit a peak sequence similarity at the level of complete genome sequences, peak sequence similarities at the level of different consensus genes often suggested alternative pairings (Figure 2).

**Figure 3:**
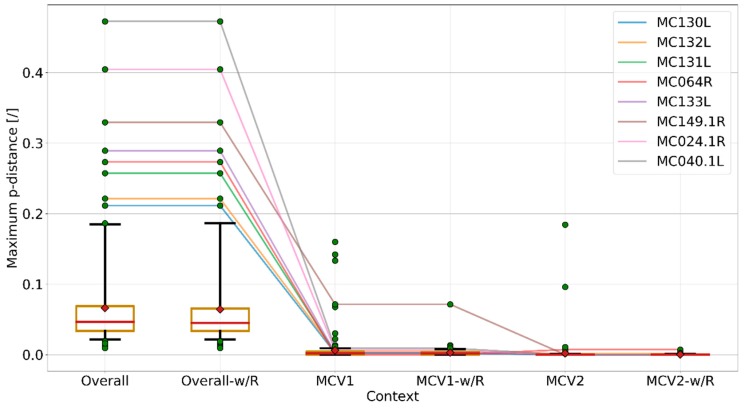
Box and whisker plots of maximum *p*-distances observed in complete gene codon multiple nucleotide sequence alignments (MSAs) and intra-genotype codon MSAs (MCV1, MCV2); “-w/R” suffixes indicate exclusion of recombinant genes. Orange boxes represent 95% CI of the median (red line), as determined by 1000 bootstrap iterations; means are shown as red diamonds. Whiskers encode the data range and extend between the fifth and 95th percentile of data; data points above or below this range are shown as green circles. Colored lines connect maximum *p*-distance points of genes that lie above the 95th percentile (recombinant genes excluded) in six different contexts. The figure indicates considerably lower *p*-distances in the intra-genotype context, compared to the overall context. Most of the anomalously high intra-genotype *p*-distances can be explained by recombination, whereas the highest *p*-distances in the overall context can mainly be attributed to MCV genotype divergence (the same genes are closely related in the intra-genotype context). The per-gene maximum dissimilarity measure suggested another possible recombination event among a known (MCV1) and unknown MCV genotype in MC149.1R (the remaining outlying point after decoupling recombination in context MCV1-w/R), although this recombination event could not be confirmed by inspection of phylogenetic trees based on nucleotide and/or codon MSAs, nor could the recombination breakpoints be elucidated by any of the recombination detection methods employed by RDP4 [53]. Visualization was carried out using the Matplotlib (v2.2.2) Python module [58].

Maximum *p*-distances of genes, measured from the codon MSAs in different contexts (Figure 3), indicate a large gap in the degrees of dissimilarity in the overall and intra-genotype contexts (Student’s *t*-test *p*-values < 1 × 10^−10^; size of each group: 164), attributing most of the dissimilarities to the inter-genotype gap. Most of the highly dissimilar outlying genes in the intra-genotype context can be explained by inter-genotype recombination (Figure 2 and Figure 3), whereas for the seemingly high mosaicity among MCV genomes of the same genotype it could be equally justified to speculate that it results from an accumulation of nucleotide substitutions during the divergence from a common ancestor (Figure 2 and Figure 3).

**Figure 4:**
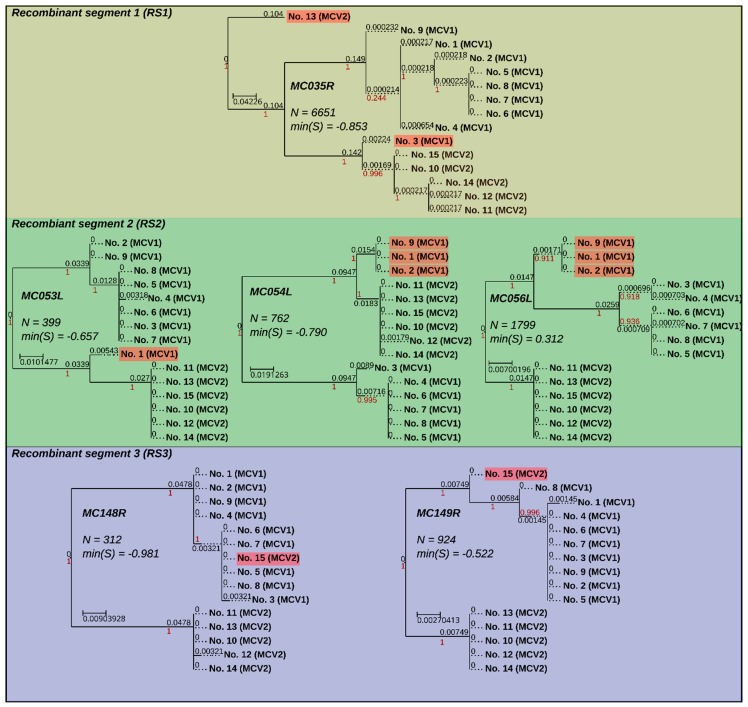
Maximum likelihood phylogenetic trees (GTR + I + G) of recombinant genes grouped according to recombinant segments. Phylogenetic trees are annotated with gene designations, lengths of gene alignments (N), and minimum values of silhouette coefficients calculated from gene alignments (min(S)). Branches are equipped with branch support values (red) and branch lengths (black). Tree branches (wherever not dotted) are metric. Sample names of recombinant end nodes are highlighted with transparent red rectangles. Phylogenetic trees were visualized using the ETE3 toolkit [47].

**Figure 5:**
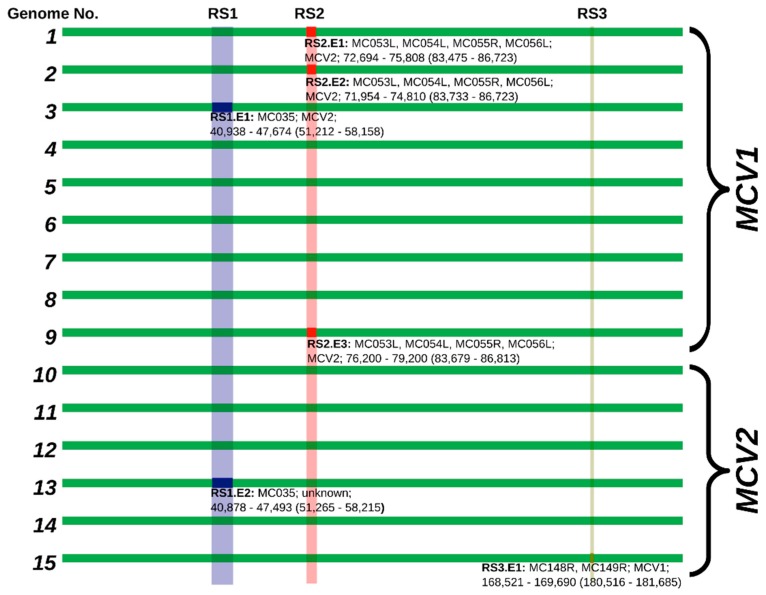
Schematic alignment of MCV genomes, depicting positions of recombinant segments. Individual recombinant segments are annotated and enumerated by position (RS1-3) and event number (in order of appearance: RS1.E1, RS1.E2, etc.). Individual recombination event annotations are structured in the following format: Recombinant segment (RS#), number of the individual event (.E#): affected genes; predicted MCV recombination donor; and location of the recombinant region in the genome (location of the recombinant region in an alignment). Semi-transparent bands indicate alignment positions of putative recombination hotspots.

Based on the analysis of silhouette coefficients, eight MCV genes (*MC006L*, *MC035R*, *MC053L*, *MC054L*, *MC056L*, *MC107L*, *MC148R*, and *MC149R*) evaluated below the set threshold and, at the same time, fulfilled the column variability criteria for putative recombinant genes. Recombination was finally confirmed with inspection of phylogenetic trees based on six genes (*MC035R*, *MC053L*, *MC054L*, *MC056L*, *MC148R*, and MC149R; Figure 4). Moreover, inspection of the wider context in the nucleotide MSAs indicated that the recombinant genes were likely transferred in three recombinant sequence segments (Figure 1, Figure 4 and Figure 5) (i) *MC035R* (Recombinant segment 1, RS1), (ii) *MC053L*, *MC054L*, and *MC056L* (RS2), and (iii) *MC148R* and *MC149R* (RS3). Nucleotide MSAs of two genes with below-threshold but positive minimum silhouette coefficient values (*MC006L* and *MC007L*) indicated truncation rather than recombination events. The truncations in the *MC006L* and *MC107L* genes affected the first approximately 2000 nt (alignment length: 4142 nt) and the last approximately 300 nt (alignment length of the gene: 1407 nt), respectively, according to the orientation of the ORFs.

To ascertain that the recombinant signals did not arise from an assembly error due to the presence of different co-infecting MCV variants, the recombinant segments were screened for intra-sample variant sites. One single nucleotide polymorphism (SNP) was found in the region corresponding to RS1 in genome No. 9. (genome No. 9: g.47699A > C; alternative allele frequency: 0.114804; local sequencing depth of coverage: 662×). The SNP represented a silent mutation at a proline amino acid site in gene *MC035R*. Although this SNP does not provide an alternative explanation for the recombinant signals identified in RS1, it may indicate the presence of MCV quasi-species in MC lesions.

The phylogenetic tree of *MC035R* (Figure 1 and Figure 4, and Figure 5: RS1) revealed the presence of two recombination events. As previously reported by López-Bueno et al. [26], it appears that an ancestor of genome No. 3 (GenBank acc. No. KY040274) obtained RS1 from a MCV2 genotype representative. The results of our study indicated the presence of one novel recombination event in RS1 of genome No. 13 (Figure 1 and Figure 4). The phylogenetic tree of RS1 suggested that the MCV2 genome No. 13 could have obtained RS1 from a so far unidentified strain of MCV, whose origin predates the divergence of MCV1 and MCV2. The upstream recombination breakpoints of RS1 were positioned at the very start of gene *MC034L* in both MCV genomes affected (Nos. 3 and 13). The positions of the downstream recombination breakpoints of RS1, on the other hand, varied slightly, they were placed at the start of gene *MC036R* and just upstream of gene *MC036R* in genomes Nos. 13 and 3, respectively.

RS2 was previously described regarding MCV genomes Nos. 1 and 2 (GenBank acc. Nos. U60315 and KY040275) [26]. In addition, herein, a new recombination within RS was observed in genome No. 9 (Figure 1 and Figure 4, and Figure 5: RS2). In the three genomes affected by recombination in RS2, the recombination breakpoints were positioned within genes *MC053L* and *MC056L*, with slight variation in their precise locations. The complete nucleotide sequence of MC054L is located between genes *MC053L* and *MC056L* and was found recombinant in all three genomes affected. Although all currently known MCV1 genomes included the *MC055R* gene, which is also positioned between genes *MC053L* and *MC056L*, but read from the complementary strand, the MCV gene MC055R is consensually absent from MCV2 genomes. Interestingly, although the RS2 in the three MCV genomes affected appears to originate from a MCV2 genotype, all three genomes retained the code for *MC055R*. Further analysis indicated that the genomes affected by recombination in RS2 (Nos. 1, 2, and 9), contained a shorter, truncated version of *MC055R* in comparison to all other currently known MCV genomes. This could suggest the existence of additional MCV variants that were not included in our study.

The results of our study suggested the existence of a novel recombinant segment RS3, which was identified in genome No. 15. RS3 spanned from 46 nt upstream of the MC148R start codon to 167 nt prior to the *MC149R* stop codon. To best of our knowledge, RS3 is the first described recombinant segment in MCV2 (Figure 1 and Figure 4, and Figure 5: RS3). The phylogenetic placement of RS3 in genome No. 15 (Figure 1 and Figure 4) suggests that the recombinant region originated from a genome of MCV1.

### 3.5. Identified Recombinant MCV Regions are Associated with Inhibition of Chemotaxis of Immune Cells and Interfering with the Host T-cell–and/or Natural Killer Cell–Related Immune Response

The three identified recombinant segments (RS1, RS2, and RS3) contained MCV genes, which are associated with viral mechanisms for evading detection by the host immune system. MC035R (RS1) is a homologue of the poxvirus B22 protein family, a group of proteins that have been shown to inactivate/prevent activation of T-cells in culture and animal models [26,66,67]. MC054L (RS2) is a secreted, poxviral homologue of the human interleukin-18-binding protein (hIL-18BP), which has been shown to antagonize gamma interferon production, and the function of T-cells and natural killer (NK) cells [68,69]. MC148R (RS3), a viral secreted CC family chemokine, was found to antagonize chemotaxis of monocytes, lymphocytes, and neutrophils, antagonizing a wide range of chemokines [4]. Interestingly, it has been noted that the *MC148R* protein products of MCV1 and MCV2 can interact with different chemokine pathways [4].

Although MC053L and MC054L (RS2) share more than 30% protein identity [4], the function of MC053L remains unclear. In a study by Xiang and Moss [68], MC053L failed to bind interleukin-18 (unlike MC054L), and the authors concluded that it may interact with another, still unidentified, ligand. To the best of our knowledge, the remaining two consensus MCV genes—affected by recombination, MC056L (RS2), a putative Zn-dependent protease involved in virion morphogenesis, similar to variola virus H1L [20], and MC149R (RS3), a putative extracellular enveloped virion protein, similar to variola A40R [20]—have not been described in relation to MCV immune evasion.

Since several immune evasion–related MCV genes were identified as recombinant, it was of interest to further analyze the intra- and inter-genotype conservation of all other known immune evasion–related MCV genes that to date have not been identified as recombinant. In addition to the MCV immune evasion genes recently reviewed by Shisler (*MC007L*, *MC066L*, *MC159L*, and *MC160L*) [4] and Chen et al. (*MC002L*, *MC006L*, *MC026L*, *MC080R*, *MC161R*, and *MC162R*) [1], MCV genes *MC005L* and *MC132L*, which were recently reported in relation to inhibition of nuclear factor kappa B [70,71], were also inspected. The nucleotide MSAs of the all MCV immune evasion–related genes listed above indicated variation. Moreover, all phylogenetic trees of the specified genes indicated higher inter-genotype phylogenetic distances in comparison to intra-genotype phylogenetic distances, which were also evident in phylogenetic trees constructed using protein MSAs, suggesting that a genotype-specific conservation of MCV immune evasion strategy may be suspected. Data regarding minimum, mean, and maximum p-distances and silhouette coefficients based on nucleotide and amino acid sequences of consensus MCV genes is provided in Supplementary Table S1A and B, respectively. Current data might indicate that the observed recombination events in MCV genomes reflect cases of successful exchange of genetic material, encoding viral immune evasion strategies between co-infecting MCVs of different genotypes. It might be speculated that the recombinant MCV genes related to immune evasion (*MC035R*, *MC054L*, and *MC148R*) increased the evolutionary fitness of the recombinant recipients at the conditions imposed by the immune systems of their respective hosts at the relevant point in time, whereas the genes not related to immune evasion (*MC053L*, *MC056L*, and *MC149R*) were horizontally transferred simply because of their proximity to the immune-related genes in the viral DNA. The latter hypothesis could also explain the variability in the detected recombination breakpoint positions.

Although the recombination detection methodologies used provided robust and sensitive means for detecting strong recombination signals, reflecting transfer of large sequence segments between different MCV genotypes, they are limited in power when only a small portion of a gene was transferred. Moreover, they do not provide direct means of evaluating intra-genotype recombination events and recombination events limited to non-coding regions. The observed intra-genotype mosaicity (Figure 2) was speculated to arise from accumulation of substitutions during colinear evolution from a common ancestry; however, a hypothesis of recombination in the intra-genotype context should not be excluded from future studies. It is important to note that both substitutions and recombinations could be reflected in similar local similarity/dissimilarity patterns at high recombination frequencies and very low lengths of horizontally transferred segments.

### 3.6. Higher Genomic Diversity among MCV1 Genomes in Comparison to MCV2 may be Explained by Their Preferred Hosts’ Immune Competence

Previous studies [11,15,19] indicated higher frequencies of MCV2 infection among patients with impaired immune response, such as patients with HIV, compared to healthy adults, which could indicate the possibility of involvement of the host T-cell response in the differences of the epidemiological distributions of MCV1 and MCV2. This appears to be consistent with the interpretation of the detected recombination events because several recombinant genes are associated with viral interference with the host T-cell response.

**Figure 6:**
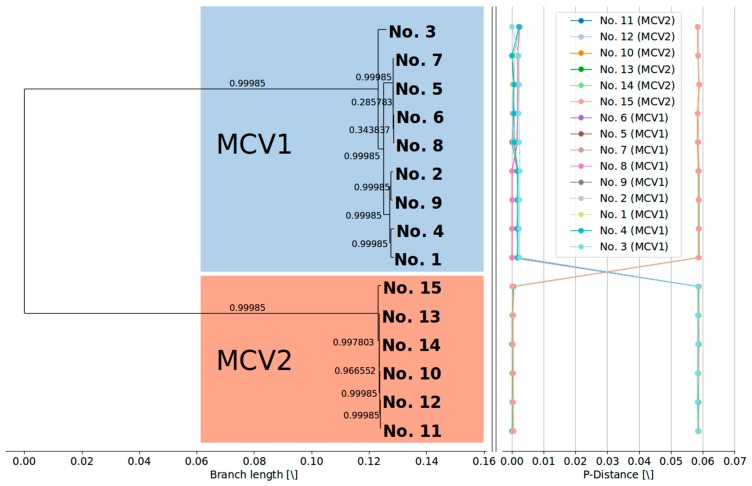
(**Left**) Maximum likelihood phylogenetic tree (GTR + I + G) with metric branch lengths and aLRT branch support values constructed based on the alignment of 15 complete MCV genome nucleotide sequences that have been stripped of the recombinant regions. (**Right**) Genome-to-genome p-distance plots after removal of identified recombinant regions, depicting a relatively large gap between the genomes of two different MCV genotypes. The phylogenetic tree was visualized using the BioPython Phylo module [57], and visualization of the pairwise p-distance plots was done using the Matplotlib (v2.2.2) Python module [58].

The identified recombination events cannot by themselves explain the complete extent of the heterogeneity of the phylogenetic branch corresponding to genotype MCV1 (Figure 6). Although the removal of the identified recombinant regions from the multiple complete genome alignment substantially reduced the mean inter-genotype *p*-distances (MCV1: 1.595 × 10^−3^ ± 0.883 × 10^−3^; MCV2: 0.297 × 10^−3^ ± 0.166 × 10^−3^), the genomic diversity of MCV1 remained approximately half an order of magnitude higher than that of MCV2. Currently available data on protein sequence variation, present among MCV1 immune evasion genes, but not among MCV2, is provided in the Supplementary Table S2.

It seems plausible that stricter selective pressures elicited by various immune-competent hosts drive the higher macro-scale diversification rate in the case of the MCV1 population, in contrast to the MCV2 population. Upon founding the infection, both MCV genotypes are expected to produce random mutations with similar mutation rates at the micro-scale. In the case of a constraining host (immune) environment, the variant with higher fitness under selective constraints could quickly become the dominant variant in the MC lesion, which would then most likely be transmitted to a new anatomical site or host. On the other hand, in the absence of explicit immunological constraints, such as an immune-compromised host, the dominant founding variant would likely remain dominant throughout the infective/reproductive cycle within an MC lesion. If the macro-scale genetic drift, which is mostly dependent on the size of the genetic bottlenecks during the transmission events, is not exceedingly high, at the macroscopic level the scenario proposed above would facilitate higher diversification among the MCV1 population relative to the population of MCV2. Complex experimental studies estimating the size of transmission bottlenecks during MCV evolution and selection coefficients of different MCV variants could further elucidate this phenomenon.

To provide a more complete understanding of MCV’s genomic landscape and evolutionary history, future analysis would need to incorporate not only North American and European MCV isolates, but also isolates from other parts of the globe. In addition, to be able to distinguish between recombination and concurrent infection with different viral strains more confidently, use of the single-virus genomic approach could be beneficial. Moreover, a deeper sampling of the MCV population, as well as generation of still missing complete genome sequences of other MCV genotypes (i.e., MCV3 and MCV4), would likely give rise to identification of novel recombination events as well as clarify the current impression on the diversity of the different MCV genotype populations.

## 4. Conclusions

This study investigated the largest collection of complete MCV genomes to date, greatly expanding the current knowledge of MCV diversity and its evolutionary landscape. Ten novel complete MCV genomes (five MCV1 and five MCV2) were sequenced, assembled, and annotated. Generation of five novel MCV2 complete genomes made possible the first investigation of the genomic diversity of MCV2. Our data suggest that MCV1 is more diverse than MCV2 and that both genotypes evolved from a common ancestor along divergent evolutionary pathways. Three recombinant segments (one novel) were identified in six MCV genomes interrogated (five in MCV1, one in MCV2); each recombinant segment included at least one viral gene associated with inhibition of chemotaxis of immune cells and/or with interference with the host’s T-cell and/or NK-cell immune responses. Recombination explains the inter-genotype mosaicity of MCV and anomalously high dissimilarities among genes of the same MCV genotype. In the context of results of previous epidemiological studies, the higher genomic diversity among MCV1 genomes in comparison to MCV2 may be explained by their preferred hosts’ immune competence.

## Figures and Tables

**Table 1:** Summary of origin, sequencing, and assembly approaches, estimated viral loads, remapping statistics, and genome characteristics of 15 MCV isolates included in the study.

No.	Viral Genotype	GenBank Acc. No.	Reference	Country of Origin	Sequencing Technique (Platform)	Assembly	Viral Load (Viral Copies/Cell)	Per-base Short Read Depth of Coverage (Mean ± SD)	Percentage of Mapped Short Reads (%)	Genome Length (nt)	ITR Length (nt)	Number of Annotated Genes
1	MCV1	U60315	Senkevich et al. [2]	Unknown	Applied Biosystems AB373A (primer-walking)	/	/	/	/	190,289	4711	178
2	MCV1	KY040275	López-Bueno et al. [26]	Spain	Illumina MiSeq (2 × 300 nt)	Short-read	/	/	/	188,253	3821	181
3	MCV1	KY040276	López-Bueno et al. [26]	Spain	Illumina MiSeq (2 × 300 nt)	Short-read	/	/	/	189,098	4252	179
4	MCV1	KY040277	López-Bueno et al. [26]	Spain	Illumina MiSeq (2 × 300 nt)	Short-read	/	/	/	188,458	3758	179
5	MCV1	MH320553	This study	Slovenia	Illumina HiSeq4000 (2 × 150 nt), ONT	Hybrid	4237	1772.92 ± 282.67	12.30	187,558	3519	177
6	MCV1	MH320552	This study	Slovenia	Illumina HiSeq4000 (2 × 150 nt), ONT	Hybrid	2527	3864.52 ± 526.58	26.11	187,884	3651	176
7	MCV1	MH320547	This study	Slovenia	Illumina HiSeq4000 (2 × 150 nt)	Short-read	1021	2243.29 ± 750.52	18.37	187,826	3559	177
8	MCV1	MH320555	This study	Slovenia	Illumina HiSeq2000 (2 × 150 nt, 2 × 250 nt), ONT	Hybrid	546,855	635.62 ± 208.74	87.98	189,292	4354	176
9	MCV1	MH320554	This study	Slovenia	Illumina HiSeq2000 (2 × 150 nt; 2 × 250 nt), ONT	Hybrid	40,351	581.67 ± 134.87	44.27	196,781	7975	175
10	MCV2	KY040274	López-Bueno et al. [26]	Spain	Illumina MiSeq (2 × 300 nt)	Short-read	/	/	/	192,183	4086	170
11	MCV2	MH320550	This study	Slovenia	Illumina HiSeq4000 (2 × 150 nt), ONT	Hybrid	26,717	2913.56 ± 417.96	18.53	196,206	7762	170
12	MCV2	MH320548	This study	Slovenia	Illumina HiSeq4000 (2 × 150 nt)	Short-read	5226	5270.58 ± 1499.21	27.27	190,319	4937	170
13	MCV2	MH320556	This study	Slovenia	Illumina HiSeq4000 (2 × 150 nt)	Short-read	4573	5861.15 ± 622.65	39.18	189,257	4319	170
14	MCV2	MH320551	This study	Slovenia	Illumina HiSeq4000 (2 × 150 nt), ONT	Hybrid	1828	3543.65 ± 546.386	24.24	192,156	5979	170
15	MCV2	MH320549	This study	Slovenia	Illumina HiSeq4000 (2 × 150 nt), ONT	Hybrid	8727	1912.23 ± 416.643	13.30	193,271	6432	170

*SD* = standard deviation, nt = nucleotides, ONT = Oxford Nanopore Technologies, ITR = inverted terminal repeats.

**Table 2:** Summary of 18 genes that were not found in either one of the 15 complete MCV genome sequences analyzed. These genes comprise approximately 10% of all MCV genes reported by Senkevich et al. [22].

Gene	Missing in Genomes (Count)	Missing in Genomes (Sequence No.)	Function/Homologues/Reference
*MC001R*	3	7, 8, 9	Predicted non-globular protein/MC164L/Senkevich et al. [20]
*MC006.1R*	6	10 *, 11, 12, 13, 14, 15	Unknown/ /Senkevich et al. [20]
*MC009.1R*	2	1 *, 4 *	Predicted non-globular protein/ /Senkevich et al. [20]
*MC009.2R*	1	1 *	Predicted non-globular protein/ /Senkevich et al. [20]
*MC017.1L*	12	3 *, 5, 6, 7, 8, 9, 8, 10 *, 11, 12, 13, 15	Predicted non-globular protein/ /Senkevich et al. [20]
*MC022.1L*	6	3 *, 5, 6, 7, 8, 9	Unknown/ /Senkevich et al. [20]
*MC042.1R*	8	1 *, 2 *, 10 *, 11, 12, 13, 14, 15	Predicted structural protein/ /Senkevich et al. [20]
*MC052R*	6	10 *, 11, 12, 13, 14, 15	Unknown/ /Senkevich et al. [20]
*MC053.1R*	13	3 *, 4 *, 5, 6, 7, 8, 9, 10 *, 11, 12, 13, 14, 15	Predicted structural protein/ /Senkevich et al. [20]
*MC053.2R*	7	4 *, 10 *, 11, 12, 13, 14, 15	Predicted C-terminal transmembrane helix/ /Senkevich et al. [20]
*MC055R*	6	10 *, 11, 12, 13, 14, 15	Unknown/ /Senkevich et al. [20]
*MC144R*	6	10 *, 11, 12, 13, 14, 15	Predicted long non-globular protein/ /Senkevich et al. [20]
*MC145.1R*	1	1 *	Predicted non-globular protein/ /Senkevich et al. [20]
*MC147R*	6	10 *, 11, 12, 13, 14, 15	Unknown/ /Senkevich et al. [20]
*MC150R*	7	6, 10 *, 11, 12, 13, 14, 15	Unknown/ /Senkevich et al. [20]
*MC152.1R*	1	3 *	Unknown/ /Senkevich et al. [20]
*MC156R*	7	6, 10 *, 11, 12, 13, 14, 15	Predicted peptide, putative secreted protein/ /NCBI Gene database
*MC164L*	9	5, 8, 9, 10 *, 11, 12, 13, 14, 15	Predicted non-globular protein/MC001R/Senkevich et al. [20]

* indicates MCV genome sequences that were available in GenBank prior to this study.

**Table 3:** Mean sample to sample *p*-distances (with and without combinatorial subsampling, balancing) between complete MCV genomes and concatenated sequences of consensus MCV genes, and GC content of the complete MCV genomes and consensus MCV genes interrogated. Fields with underlined boldface text indicate mean distance centroid sequences (minimum mean *p*-distance to all other MCV genomes/concatenated consensus genes).

		Mean *p*-Distances						GC Content	
		Sample vs. All				Intra-Genotype			
Number	Viral Genotype	Genome	Genome (Balancing)	Consensus Genes	Consensus Genes (Balancing)	Genome	Consensus Genes	Genotype	Consensus Genes
1	MCV1	0.02821 ± 0.02885	0.03500 ± 3.3 × 10^−4^	0.02490 ± 0.02582	0.03100 ± 3.0 × 10^−4^	0.002909 ± 2.546 × 10^−3^	0.002314 ± 2.521 × 10^−3^	0.6336	0.6435
2	MCV1	0.02793 ± 0.02877	0.03471 ± 3.2 × 10^−4^	0.02484 ± 0.02595	0.03100 ± 3.0 × 10^−4^	0.002730 ± 2.493 × 10^−3^	0.002158 ± 2.497 × 10^−3^	0.6342	0.6333
3	MCV1	0.02954 ± 0.02465	0.03535 ± 9 × 10^−5^	0.02690 ± 0.02138	0.03193 ± 5 × 10^−5^	0.007318 ± 2.658 × 10^−3^	0.007500 ± 2.675 × 10^−3^	0.6338	0.6430
4	MCV1	0.02827 ± 0.02966	0.03526 ± 2.5 × 10^−4^	0.02504 ± 0.02642	0.03127 ± 3.0 × 10^−4^	0.002317 ± 1.959 × 10^−3^	0.001969 ± 2.675 × 10^−3^	0.6345	0.6433
5	MCV1	0.02822 ± 0.02991	0.03527 ± 3.2 × 10^−4^	0.02497 ± 0.02657	0.03123 ± 3.0 × 10^−4^	0.002107 ± 2.390 × 10^−3^	0.001795 ± 2.418 × 10^−3^	0.6341	0.6431
6	MCV1	0.02824 ± 0.02987	0.03528 ± 3.2 × 10^−4^	0.02500 ± 0.02660	0.03127 ± 3.0 × 10^−4^	0.002152 ± 2.411 × 10^−3^	0.001794 ± 2.417 × 10^−3^	0.6339	0.6432
7	MCV1	0.02824 ± 0.02989	0.03529 ± 3.2 × 10^−4^	0.02512 ± 0.02658	0.03138 ± 3.0 × 10^−4^	0.002140 ± 2.408 × 10^−3^	0.001919 ± 2.440 × 10^−3^	0.6340	0.6431
8	MCV1	0.02823 ± 0.02982	0.03526 ± 3.3 × 10^−4^	0.02496 ± 0.02657	0.03122 ± 3.0 × 10^−4^	0.002181 ± 2.445 × 10^−3^	0.001785 ± 2.412 × 10^−3^	0.6332	0.6430
9	MCV1	0.02798 ± 0.02881	0.03477 ± 3.3 × 10^−4^	0.02482 ± 0.02598	0.03094 ± 3.0 × 10^−4^	0.002736 ± 2.547 × 10^−3^	0.002122 ± 2.508 × 10^−3^	0.6312	0.6434
10	MCV2	0.03999 ± 0.02877	0.03415 ± 2.3 × 10^−4^	0.03552 ± 0.02551	0.03035 ± 2.0 × 10^−4^	0.001233 ± 2.271 × 10^−3^	0.001168 ± 2.410 × 10^−3^	0.6432	0.6524
11	MCV2	0.04005 ± 0.02877	0.03421 ± 2.3 × 10^−4^	0.03557 ± 0.02554	0.03039 ± 2.0 × 10^−4^	0.001263 ± 2.256 × 10^−3^	0.001173 ± 2.408 × 10^−3^	0.6403	0.6524
12	MCV2	0.04002 ± 0.02881	0.03418 ± 2.4 × 10^−4^	0.03557 ± 0.02554	0.03040 ± 2.0 × 10^−4^	0.001223 ± 2.274 × 10^−3^	0.001177 ± 2.415 × 10^−3^	0.6438	0.6523
13	MCV2	0.04271 ± 0.02710	0.03721 ± 9 × 10^−5^	0.03866 ± 0.02389	0.03380 ± 7 × 10^−5^	0.005307 ± 2.379 × 10^−3^	0.005506 ± 2.464 × 10^−3^	0.6441	0.6518
14	MCV2	0.04002 ± 0.02880	0.03418 ± 2.4 × 10^−4^	0.03557 ± 0.02554	0.03039 ± 2.0 × 10^−4^	0.001231 ± 2.263 × 10^−3^	0.001165 ± 2.409 × 10^−3^	0.6424	0.6523
15	MCV2	0.04004 ± 0.02850	0.03426 ± 2.3 × 10^−4^	0.03560 ± 0.02539	0.03045 ± 2.0 × 10^−4^	0.001580 ± 2.314 × 10^−3^	0.001365 ± 2.4426 × 10^−3^	0.6414	0.6523

The data dispersion term is given as standard deviation.

## Supplementary Materials

The following are available online at http://www.mdpi.com/1999-4915/10/11/586/s1, Supplementary Table S1A: Nucleotide p-distance summary of MCV genes with corresponding values of silhouette coefficients, Supplementary Table S1B: Amino acid p-distance summary of MCV genes with corresponding values of silhouette coefficients and Supplementary Table S2: MCV1 immune evasion gene specific protein sequence variation.
